# The contemporary role of Impella in a comprehensive mechanical circulatory support program: a single institutional experience

**DOI:** 10.1186/s12872-015-0119-9

**Published:** 2015-10-14

**Authors:** Marina Pieri, Rachele Contri, Dario Winterton, Matteo Montorfano, Antonio Colombo, Alberto Zangrillo, Michele De Bonis, Federico Pappalardo

**Affiliations:** Department of Anesthesia and Intensive Care, IRCCS San Raffaele Scientific Institute, Via Olgettina 60, 20132 Milan, Italy; Department of Interventional Cardiology, IRCCS San Raffaele Scientific Institute, Via Olgettina 60, 20132 Milan, Italy; Department of Cardiac Surgery, IRCCS San Raffaele Scientific Institute, Via Olgettina 60, 20132 Milan, Italy

**Keywords:** Mechanical circulatory support, Cardiogenic shock, IMPELLA, ECMO

## Abstract

**Background:**

The treatment of cardiogenic shock with percutaneous mechanical circulatory support (MCS) is attractive: however, at present it is not clear which is the best strategy, as no survival benefit has been demonstrated for any device as single therapy. Aim of this study is to describe the role of percutaneous Impella in a comprehensive MCS program.

**Methods:**

Observational study on 22 patients supported with the Impella device in our hospital from May 2013 to June 2014.

**Results:**

Four patients (18 %) were treated with Impella alone, 8 patients (36 %) were treated with Impella and IABP, 6 patients (27 %) with Impella and VA ECMO, and 4 patients (18 %) with Impella, IABP and VA ECMO.

The cause of cardiogenic shock was myocardial infarction (CSMI) in 9 patients (41 %), postcardiotomic cardiogenic shock in 5 (23 %), and a miscellaneous of other causes in the remaining 8 (36 %). Eight Impella devices (36 %) were placed under transesophageal echocardiographic guidance, while 14 (64 %) under fluoroscopy. The device was removed with manual compression at bedside and no vascular complications were observed.

Duration of Impella support was 107 (54–141) hours and duration of ventilation was 48 (14–92) hours. Hemolysis occurred in 6 patients (27 %), while major bleeding in 4 patients (18 %). Survival was 73 %: 13 patients (58 %) showed recovery of cardiac function; 1 patient (5 %) was bridged to left ventricular assist device (LVAD) implantation, 1 patient (5 %) to heart transplantation (HTx) and 1 patient (5 %) received a BiVAD and was eventually bridged to HTx.

**Conclusions:**

Our data suggest that a multi-device approach, encompassing active LV support with Impella, is safe and can significantly improve survival in patients with cardiogenic shock.

## Background

Mechanical circulatory support (MCS) is considered the standard of care in the treatment of refractory cardiogenic shock (CS). The availability of temporary left and biventricular assist devices, placed either percutaneously or surgically, has widened the therapeutic armamentarium beyond the use of intraaortic balloon pump counterpulsation (IABP) and venoarterial extracorporeal membrane oxygenation (VA ECMO).

In this context, the Impella device (ABIOMED Inc, Danvers, MA) has gained extraordinary diffusion, because it is safe, easy to use and its efficacy has been proven in many clinical scenarios [1–5].

Impella technology comprises Impella 2.5, the Impella CP, and Impella 5.0/LD devices, which can generate up to 2.5 L/min, 4 L/min, and 5.0 L/min of forward flow in the systemic circulation, respectively [6, 7]. Although strong evidence exists regarding the efficacy of MCS devices in the treatment of cardiogenic shock, a combined multidevice approach is not commonly reported.

The CS patient population suffering from refractory cardiogenic shock is extremely heterogeneous in terms of etiology of CS (e.g., myocardial infarction, postcardiotomic shock, out-of hospital cardiac arrest, myocarditis) and careful tailoring of therapeutic options is mandatory. The concomitant use of different devices may overcome the limitations of each form of support.

Although the treatment of cardiogenic shock with MCS plays a crucial role in clinical practice, the best strategy is still uncertain, as no survival benefit has been demonstrated for any device as the sole therapy so far [8]. Aim of this study is to describe the role of percutaneous Impella device in the contemporary MCS armamentarium.

## Methods

The present study is in compliance with the Helsinki Declaration. After approval by the local ethical committee (“Ospedale San Raffaele Ethical Committee”), we performed a prospective observational study of all the patients treated with Impella devices in the Cardiac Intensive Care Unit of IRCCS San Raffaele Scientific Institute from May 2013 to June 2014. The patients in our Department signed a written consent for the use of their data for scientific purpose. No specific written consent was obtained since all patients’ data were anonymized and de-identified prior to analysis.

All patients received either the 2.5 Impella device or CP Impella device. The Impella device was inserted either under fluoroscopy in the cath lab, or at bedside under transesophageal (TE) echocardiographic guidance, according to the clinical situation. The Impella pump speed was set to optimally unload the LV as assessed with transesophageal echocardiography.

Removal of the Impella device was performed percutaneously, with manual compression of the access site for at least 45 min. General baseline information, data on mechanical circulatory support and its duration, together with the outcomes were recorded for all patients. Hemodynamic and echocardiographic data were collected for the study population at 5 time points: before Impella implantation, 30 min after implantation, 24 h after implantation, at weaning (i.e. recorded in the 24 h before device removal), 24 h after device discontinuation.

Some patients received concomitant mechanical circulatory support with IABP and/or VA ECMO. The ECMO circuit setup included a centrifugal pump and a coated polymethylpentene oxygenator. In case of peripheral cannulation, outlet cannulas ranged from 21 to 29 French, and inlet cannulas from 15 to 19 French. Patients undergoing central VA ECMO cannulation had an outlet cannula of 32–34 French, and an inlet cannula of 20–24 Fr. A distal perfusion cannula was placed whenever possible in all patients undergoing peripheral VA ECMO (range 5–8 French) to prevent leg ischemia. Patients who needed intravenous anticoagulation were administered bivalirudin titrated to an activated partial thromboplastin time (aPTT) between 45 and 60 s. In the absence of contraindications, the purge fluid of Impella device also contained unfractionated heparin (either 25U/ml or 50 U/ml) according to clinical need. Hemolysis was defined as an increase in serum LDH levels above 1000 U/l associated with an increase in free plasma hemoglobin above the upper limit of laboratory range in at least 2 consecutive blood samples within 24 h. Major bleeding was defined as intracranial, intraocular, retropharyngeal or retroperitoneal bleeding requiring either radiological intervention or surgical revision or decrease in serum hemoglobin >3 g/dl or the need for transfusion of at least two packed red blood cell units.

Inotropic score (IS) was calculated as: (dopamine μg/kg/min × 1) + (dobutamine μg/kg/min × 1) + (milrinone μg/kg/min × 15) + (epinephrine/norepinephrine μg/kg/min x 100) [9]. Vascular complications included access site or access-related vascular injury requiring unplanned percutaneous or surgical intervention, and distal embolization.

In the absence of a standardized definition, right ventricular dysfunction was diagnosed if at least 2 out of the following criteria were present at echocardiographic evaluation: severe tricuspid valve regurgitation, end diastolic right ventricular diameter ≥35 mm, a value of tricuspid annular plane systolic excursion (TAPSE) <1.5 cm, a value of sTDI of tricuspid anulus <10 cm/sec and poor ejection fraction.

### Statistical analysis

Categorical variables are reported as numbers (percentages), whereas continuous variables are expressed as mean ± standard deviation and median with interquartile range (25^th^-75^th^ percentile). The analysis of variance (ANOVA) was used to estimate the time interaction for each variable. Data were analyzed using Microsoft Excel 2007 (Microsoft Office 2007, Redmon, WA, US) and SAS 9.2 (SAS Institute Inc. Cary, NC, USA).

## Results

Twenty-two patients received mechanical circulatory support with Impella during the study period. Baseline characteristics of the patients are shown in Table 1. Twenty patients (90 %) were male. Mean age was 60 ± 13 years. As shown in Table 2, the cause of cardiogenic shock was myocardial infarction in 9 patients (41 %), and postcardiotomic cardiogenic shock in 5 (23 %). Indication to Impella implantation, together with MCS-related management and outcomes can be found in Table 2. The outcomes of the whole population are further presented in the Table 3. Nineteen patients (86 %) were treated with Impella 2.5, while the 3 remaining patients (14 %) received Impella CP.

**Table 1:** Baseline data

Parameter	Value
Male, *n*	20 (90)
Age, years	60 ± 13, 62 (52–68)
Height, cm,	172 ± 7, 175 (170–178)
Weight, kg	74 ± 11, 75 (70–84)
BSA	1.9 ± 0.1, 1.9 (1.8–2)
BMI	25 ± 3, 24.8 (22.9–26.2)
Comorbidity	
Hypertension, *n*	6 (22)
Chronic renal failure, *n*	4 (18)
Dislipidemy, *n*	4 (18)
Overweight, *n*	3 (14)
Type II diabetes, *n*	1 (4.5)
Coronary artery disease, *n*	8 (36)
Idiopatic dilatative cardiomyopathy, *n*	2 (9)
Chronic obstructive pulmonary disease, *n*	3 (14)
Hypothyroidism, *n*	1 (4.5)
None, *n*	5 (22)
Cause of cardiogenic shock	
Acute myocardial infarction, *n*	9 (41)
Postcardiotomic, *n*	5 (23)
Other, *n*	8 (36)

Data shown as number (percentage) or as mean ± standard deviation and median (interquartile range)

*BSA*–Body surface area; BMI–Body mass index

**Table 2:** Indication to IMPELLA , MCS-related management and outcomes

Patient ID	Indication to implantation	Rationale for impella’s implantation	Devices implanted	Hemolysis	Anticoagulation	Major bleeding	Outcome
IMP1	CSMI (STEMI)	Circulatory support, LV venting	IMPELLA 2.5, IABP	No	UFH 25 U/ml (purge fluid)	No	LVAD
IMP2	Cardiogenic shock (arrhytmia)	LV venting during ECMO in severe AI	IMPELLA 2.5, VA ECMO	No	UFH 25 U/ml (purge fluid)	No	Bridge to BIVAD and HTX
IMP3	CSMI (STEMI)	Coronary perfusion, LV venting during ECMO	IMPELLA 2.5, VA ECMO	No	IV bivalirudin	No	Organ donor (brain death)
IMP4	Cardiogenic shock	LV venting during ECMO	IMPELLA 2.5, VA ECMO	No	IV bivalirudin	No	Recovery
IMP5	Postcardiotomic cardiogenic shock (Sleeve procedure)	Coronary perfusion, LV venting	IMPELLA 2.5, VA ECMO, IABP	Yes	IV bivalirudin	Yes	Bridge to HTX
IMP6	CSMI (STEMI)	Coronary perfusion, circulatory support	IMPELLA 2.5, IABP	No	IV bivalirudin	No	Recovery
IMP7	Cardiogenic shock following percutaneous VT ablation	Circulatory support, LV venting	IMPELLA 2.5	No	IV bivalirudin	No	Recovery
IMP8	Cardiogenic shock (coronary ischemia)	Circulatory support	IMPELLA CP, IABP	No	UFH 50 U/ml (purge fluid)	Yes	Death (MOF-withdrawal of care)
IMP9	CSMI (STEMI)	Coronary perfusion, LV venting	IMPELLA 2.5, VA ECMO	No	-	No	Death (MOF-withdrawal of care)
IMP10	CSMI (STEMI)	Coronary perfusion, circulatory support, LV venting	IMPELLA 2.5, IABP	No	UFH 50 U/ml (purge fluid)	No	Recovery
IMP11	CSMI (STEMI)	Coronary perfusion, circulatory support	IMPELLA 2.5, IABP	No	UFH 50 U/ml (purge fluid)	No	Recovery
IMP12	Postcardiotomic cardiogenic shock (LV aneurismectomy )	LV venting	IMPELLA 2.5, VA ECMO	Yes	IV bivalirudin	Yes	Death (progressive cardiogenic shock)
IMP13	Postcardiotomic cardiogenic shock (CABG)	Coronary perfusion, circulatory support	IMPELLA CP, IABP	No	UFH 50 U/ml (purge fluid)	No	Recovery
IMP14	CSMI (STEMI)	Coronary perfusion, LV venting	IMPELLA 2.5, VA ECMO	Yes	IV bivalirudin	No	Death (MOF-withdrawal of care)
IMP15	Postcardiotomic cardiogenic shock (Mitral plasty)	Coronary perfusion, circulatory support, LV venting	IMPELLA 2.5, VA ECMO, IABP	No	IV bivalirudin	No	Recovery
IMP 16	CSMI (STEMI)	Coronary perfusion, circulatory support	IMPELLA 2.5, IABP	No	IV bivalirudin	No	Recovery
IMP 17	Cardiogenic shock (cronic myocarditis)	Coronary perfusion, circulatory support	IMPELLA 2.5, IABP	Yes	IV bivalirudin	No	Recovery
IMP18	Postcardiotomic cardiogenic shock (CABG)	LV venting during VA ECMO	IMPELLA 2.5, VA ECMO, IABP	No	UFH 25 U/ml (purge fluid)	Yes	Recovery
IMP 19	Cardiogenic shock (arrhytmia)	Coronary perfusion, circulatory support, severe AI	IMPELLA 2.5	No	IV bivalirudin	No	Recovery
IMP 20	Cardiogenic shock (coronary artery disease)	Coronary perfusion, circulatory support	IMPELLA CP	No	IV bivalirudin	No	Recovery
IMP 21	Cardiogenic shock (chronic heart failure, multiple valvulopathy)	LV venting during VA ECMO	IMPELLA 2.5, VA ECMO, IABP	Yes	IV bivalirudin, UFH 25 U/ml (purge fluid)	No	Death (MOF-withdrawal of care)
IMP 22	CSMI (STEMI)	Coronary perfusion, circulatory support, moderate AI	IMPELLA CP	Yes	UFH 25 U/ml (purge fluid)	No	Recovery

*CSMI* – cardiogenic shock myocardial infarction; *STEMI* – ST elevation myocardial infarction; *VT* – ventricular tachycardia; *LV* – left ventricle; *VA ECMO* – venoarterial extracorporeal membrane oxygenation; *IABP* – intraortic balloon pump; *IV* – intravenous; *UFH* – unfractionated heparin; *VAD*–ventricular assit device; *HTX* – heart transplantion

**Table 3:** Outcome data

Parameter	Value
Impella support duration , hours	107 (54–141), 105 ± 58
Mechanical ventilation, hours	48 (14–92), 92 ± 153
Intensive care unit stay, days	8 (4–11), 13 ± 17
Hospital stay, days	14 (8–30), 18 ± 17
Serum troponin peak, ng/ml	8002 (1111–17613), 15953 ± 26189
Hemolysis, *n*	6 (27)
Major bleeding, *n*	4 (18)
Thromboembolic complications, *n*	0 (0)
Vascular complications, *n*	0 (0)
Arrhythmia, *n*	1 (5)
Lesions of cardiac structure, *n*	0 (0)
Survival, *n*	16 (73)
Outcomes	
Recovery, *n*	13 (58)
LVAD implantation, *n*	1 (5)
HTX, *n*	2 (9)
Organ donation, *n*	1 (5)
Death, *n*	5 (23)

Data shown as number (percentage) or as median (interquartile range) and mean ± standard deviation

*LVAD* – Left ventricular assist device; *HTX* – Heart transplantation

Four patients (18 %) were treated with Impella alone, while 18 patients underwent Impella support in the context of a multidevice strategy: 8 patients (36 %) were treated with Impella on top of IABP, 6 patients (27 %) with Impella and VA ECMO (Impella was placed within the first 24 h as active left ventricular venting), and 4 patients (18 %) with Impella, IABP, and VA ECMO.

Of these 4 patients, 3 had central VA ECMO and 1 peripheral VA ECMO. Two patients had severe and 3 had moderate aortic regurgitation at the time of Impella placement. Eight Impella devices (36 %) were inserted at bedside under TE echocardiography, while 14 (64 %) in the catheterisation laboratory under fluoroscopic guidance. Success rate of implantation was 100 % and no device failure was observed. Device removal was performed with manual compression at bedside; we do not report any vascular complications. Median duration of Impella support was 107 (54–141) hours.

Hemolysis was observed in 6 patients (27 %). Major bleeding occurred in 4 patients (18 %): 2 cases of gastrointestinal bleeding and 2 cases of cardiac tamponade after cardiac surgery. No patient who received isolated Impella support experienced bleeding. None of the bleedings could be attributed to the Impella device. We do not report any episode of Impella-related vascular complication. One episode (5 %) of Impella-related ventricular arrhythmia was recorded. No neurological complications was recorded. Sixteen patients (73 %) received mechanical ventilation during support, while 6 patients (27 %) were in spontaneous breathing during the whole period of mechanical circulatory support. Median duration of mechanical ventilation was 48 (14–92) hours.

Four patients (18 %) suffered from acute renal failure requiring continuous venovenous hemofiltration (CVVH), and one patient with chronic liver insufficiency required molecular adsorbent recirculation system (MARS) during Impella support.

Overall survival was 73 %: 13 patients (58 %) experienced recovery of heart function, 1 patient (5 %) was bridged to LVAD implantation, 1 patient (5 %) underwent heart transplantation, and 1 patient (5 %) was implanted a BiVAD and was bridged to heart transplantation. Five patients (22 %) died, and 1 patient (5 %) met the criteria for organ donation. Overall intensive care unit stay was 8 (4–11) days, and hospital stay was 14 (8–30) days. Hemodynamic and clinical data recorded at the 5 different time points are shown in Table 4: notably, the improvement of ejection fraction and mixed SVO_2_ during Impella support were statistically significant (*p* = 0.042 and *p* = 0.022, respectively). No other parameter, including lactates, improved significantly during Impella support.

**Table 4:** Hemodynamic and clinical data at the different time points: baseline, 30 min after Impella implantation, 24 h after implantation, before weaning, 24 h after weaning

Parameter	Baseline	30 min	24 h	Before Weaning	24 h after weaning	P
Inotropic score, *n*	5 (0–20)	10 (5–20)	5 (0–6)	0 (0–4)	0 (0–4)	0.1
Ejection fraction , %	20 (11.2–25)	15 (10–45)	20 (12–29)	32 (25–45)	32 (25–45)	0.042
LV-EDD, mm	55 ± 14.9	51 ± 13.6	51 ± 7.6	49 ± 11.2	59 ± 11.2	0.7
Cardiac Index, L/min/m2	1.8 (1.3–2.6)	2.5 (1.8–3.3)	2.3 (1.8–2.6)	2.6 (2.2–2.8)	2.6 (2.2–2.8)	0.7
Systolic pressure, mmHg	114 ± 29.9	115 ± 25.9	118 ± 17.6	112 ± 25.3	112 ± 25	0.8
Mean arterial pressure, mmHg	79 ± 11.1	82 ± 12.6	77 ± 13.3	78 ± 16.4	78 ± 16.4	0.3
Wedge Pressure, mmHg	15 ± 4.2	16 ± 2.5	13 ± 4.2	14 ± 3	14 ± 3.0	0.3
Lactates, mmol/L	1.5 (1.4–11)	5.7 (2.2–7.6)	2.3 (1.6–7.4)	1 (1.0–1.7)	1.1 (1.0–1.7)	0.5
Pro-BNP, pg/ml	15102 (7847–222422)	-	12541 (1812–20344)	6488 (3319–18829)	6488 (3319–18829)	0.1
Mixed SVO^2^, %	58 ± 9.6	65 ± 12.5	62 ± 11.6	69 ± 2.5	69 ± 2.5	0.022

Data shown as number (percentage) or as median (interquartile range)

*LV-EDD* – Left ventricular end diastolic diameter; *BNP* – Brain natriuretic peptide

## Discussion

Despite significant improvements in coronary intervention techniques, new frontiers in antithrombotic regimens and significant advances in cardiac intensive care, mortality of cardiogenic shock remains unacceptably high (over 40 %) [8–10].

Our study reports an improved short-term survival and a low rate of complications with the use of Impella 2.5/CP in the setting of cardiogenic shock of various etiologies. The purpose of our study was to evaluate the application of the percutaneous Impella device 2.5 and CP either as the sole form of support or as part of a combined MCS strategy with either IABP or VA ECMO in the setting of cardiogenic shock of different etiologies (CSMI, postcardiotomic cardiogenic shock, myocarditis and cardiogenic shock of other etiologies) in a population of 22 patients treated in our intensive care unit over a 1-year period.

In our study the availability of a percutaneous LV assist device has proved to be a valuable tool as adjunct to other MCS devices. Indeed, the Impella pump can be used as LV support device alone or in combination with IABP and VA ECMO. In our case series, the main indication (i.e. in the Impella alone group) was in the presence of moderate signs of poor anterograde flow combined with severe pulmonary congestion. The latter shows the beneficial effects of active LV venting in the “stone heart” with or without aortic regurgitation during VA ECMO.

VA-ECMO increases LV afterload secondary to retrograde blood flow. In a severely dysfunctional heart with a normal aortic valve, the increased afterload prevents aortic valve opening, leading to LV volume overload and increased wall stress, and therefore to pulmonary venous congestion, pulmonary vascular injury and stasis with thrombus formation within the LV cavity [11, 12].

Effective LV venting (as confirmed by clearing of lung fields and reduction in LV dimensions) is crucial. Boulate et al. [13] showed that about a third of patients undergoing VA-ECLS bridge to LVAD suffer post-implant acute lung injury (ALI), which carries a high mortality rate at 60 days follow up (87 %) and that one of the main risk factors for ALI is the presence of pulmonary edema (i.e. incomplete LV unloading) while under ECLS preceding LVAD implantation. Current management of LV distension during VA ECMO includes IABP, percutaneous atrial septostomy with a catheter placed into the left atrium, and central ECMO cannulation with direct placement of the inflow cannulae in the left atrium or LV. However, such LV venting techniques have not been systematically validated and results of our series are strikingly impressive.

A peripheral VA ECMO configuration was adopted in the vast majority of patients (19 cases), as it is more versatile, can be performed at bedside, and is applicable to different clinical scenarios. On the contrary central VA ECMO must be inserted in the operating theatre by a cardiac surgeon, and was performed in only 3 patients suffering from postcardiotomic cardiogenic shock . No switching from peripheral to central VA ECMO cannulation was performed.

The strategy of direct venting the LV chamber in postcardiotomic patients instead of using Impella is attractive. However, Impella was inserted in the operating room at the end of surgery only in 2 postcardiotomic patients of our study population, while in the other 3 cases the Impella device was implanted in the ICU within the first 12 postoperative hours, in the presence of signs of inadequate LV unloading and stasis in the left ventricle. Surgical venting is successful but can be positioned only in the operating room. On the contrary, the Impella pump can be inserted even after surgery in the ICU. For this reason, the availabilty of a percutaneous device for LV unloading is of fundamental importance also in patients with central VA ECMO. For the same reason, before the availability of Impella at our Institution, IABP was the most applied method to unload the LV, with surgical venting being performed only in a few cases.

Furthermore, support with the Impella pump can be started as an adjunct to aortic counterpulsation in patients failing on IABP or in patients with profound cardiogenic shock and severe LV overload as observed in the AMI-related CS cohort. In our study, most patients experienced either myocardial recovery (58 %) or were bridged to LVAD or to heart transplantation (14 %). Mortality rate was 23 %. One patient (5 %) met the criteria for organ donation. Eligibility for organ donation was considered as the result of satisfactory organ perfusion and therefore efficacy of the MCS combination applied (VA ECMO and Impella 2.5). Brain death was explained as the consequence of a prolonged no flow or low flow phase that preceded ALS arrival and MCS implantation.

Moreover, we describe a low complication rate. This observation is mainly the result of a low incidence of major bleeding events: 2 cases of gastrointestinal bleeding and 2 cases of cardiac tamponade after cardiac surgery. However, it must be stressed that no patient who received isolated Impella support experienced bleeding. In addition, such events were observed either in patients who received the VA ECMO-Impella combination after cardiac surgery and therefore were managed with high dose systemic anticoagulation or in patients who received VA ECMO and combined Impella placement in the setting of late referral cardiogenic shock after multiorgan failure (MOF), including liver failure, had ensued.

No vascular complications were observed at the femoral access site. Both the Impella 2.5 (Introducer size 13 Fr) and CP (Introducer size 14 Fr) were removed safely by manual compression. Impella was inserted at the patient’s bedside in six cases (27 %) under transesophageal (TEE) guidance. Such data is of uttermost importance and has extraordinary potential clinical implications. Indeed, timing seems to be a key factor in improving the survival of patients with CS. The duration of the low output phase is indeed the main determinant of multiple organ failure which might become irreversible even after the initial cause of shock has been treated and tissue perfusion restored. In this context, the feasibility of TE echocardiography-guided Impella placement is not only beneficial in terms of restoration of adequate perfusion and LV venting, but also reduces implantation time. Moreover, it overcomes the need to transfer a critical patient on extracorporeal support to the cath lab for device insertion under fluoroscopic guidance.

This study has some limitations. Firstly, the study population is small and there is no control group. Nonetheless, we consider our results impressive, as we describe a survival rate (73 %), that had never been reported in literature in this patient population. Furthermore, this survival rate does not include the patient (1 case, 5 % of the population) who was considered eligible for organ donation as a consequence of satisfactory organ perfusion as achieved through MCS implantation. Secondly, indication for MCS support was established at the discretion of the attending physician. Furthermore, the non-randomized design of the study does not allow to establish clear indications on the application of this therapeutic strategy and further studies are needed for this purpose.

## Conclusions

Our preliminary data suggest that the best strategy to increase the survival of cardiogenic shock in the present era can not be relied upon a “one size fits all” strategy and we have shown that systematic application of LV venting with Impella pump strikingly increases the survival rate of cardiogenic shock patients. The feasibility of TE echocardiography-guided Impella placement at the ICU bedside might further expand the application of this strategy. Despite the highly invasive nature of the CS management, patients experienced very few complications.

Mortality of cardiogenic shock has not improved over the last decades, despite the systematic use of different circulatory support devices. The design of previous studies has consistently focused on the comparison of one device versus another or versus medical treatment. Even if not physiopathologically sound, this approach is effective and needs further studies.

## Abbreviations

ALI: Acute lung injury
ALS: Advanced life support
APTT: Activated partial tromboplastin time
CSMI: Cardiogenic shock myocardial infarction
HTX: Heart transplantation
IS: Inotropic score
LVAD: Left ventricular assist device
MCS: Mechanical circulatory support
MOF: Multiorgan failure
IABP: Intraaortic balloon pump
TAPSE: Tricuspid anular plane systolic excursion
TEE: Trans-esophageal echocardiography
VA ECMO: Venoarterial extracorporeal membrane oxygenation
